# The highest-copy repeats are methylated in the small genome of the early divergent vascular plant *Selaginella moellendorffii*

**DOI:** 10.1186/1471-2164-9-282

**Published:** 2008-06-12

**Authors:** Agnes P Chan, Admasu Melake-Berhan, Kimberly O'Brien, Stephanie Buckley, Hui Quan, Dan Chen, Matthew Lewis, Jo Ann Banks, Pablo D Rabinowicz

**Affiliations:** 1 J. Craig Venter Institute (JCVI), Rockville, MD 20850, USA; 2 Department of Botany and Plant Pathology, Lilly Hall, Purdue University, West Lafayette, IN 47907, USA; 3 Institute for Genome Sciences, University of Maryland School of Medicine, Baltimore, MD 21201, USA; 4 Program in Oncology, University of Maryland School of Medicine, Baltimore, MD 21201, USA; 5 Applied Biosystems, 2130 Woodward St., Austin, TX 78744, USA; 6 Department of Biochemistry and Molecular Biology, University of Maryland School of Medicine, Baltimore, MD 21201, USA

## Abstract

**Background:**

The lycophyte *Selaginella moellendorffii *is a vascular plant that diverged from the fern/seed plant lineage at least 400 million years ago. Although genomic information for *S. moellendorffii *is starting to be produced, little is known about basic aspects of its molecular biology. In order to provide the first glimpse to the epigenetic landscape of this early divergent vascular plant, we used the methylation filtration technique. Methylation filtration genomic libraries select unmethylated DNA clones due to the presence of the methylation-dependent restriction endonuclease McrBC in the bacterial host.

**Results:**

We conducted a characterization of the DNA methylation patterns of the *S. moellendorffii *genome by sequencing a set of *S. moellendorffii *shotgun genomic clones, along with a set of methylation filtered clones. Chloroplast DNA, which is typically unmethylated, was enriched in the filtered library relative to the shotgun library, showing that there is DNA methylation in the extremely small *S. moellendorffii *genome. The filtered library also showed enrichment in expressed and gene-like sequences, while the highest-copy repeats were largely under-represented in this library. These results show that genes and repeats are differentially methylated in the *S*. *moellendorffii *genome, as occurs in other plants studied.

**Conclusion:**

Our results shed light on the genome methylation pattern in a member of a relatively unexplored plant lineage. The DNA methylation data reported here will help understanding the involvement of this epigenetic mark in fundamental biological processes, as well as the evolutionary aspects of epigenetics in land plants.

## Background

DNA methylation has been found throughout the plant kingdom, typically in cytosines, forming part of symmetric (CpNpG and CpG) and asymmetric (CpNpN) sites [1,2]. The proportion of methylated cytosine in plants is variable, ranging from 6% in *Arabidopsis *[3] to 25% in maize [4]. DNA methylation has been associated with the inactivation of transposons and silencing of genes [5-10], and it has also been proposed that the function of DNA methylation is to decrease transcriptional "noise" [11].

In plants, most DNA methylation is found in repetitive elements, while genes and other low copy sequences are generally hypomethylated [12-17].

Because of the large size of many plant genomes, particularly those of important crops [18], gene-enriched sequencing strategies have been designed as an alternative to whole genome sequencing in an attempt to capture the so-called gene-space of such genomes. One of these gene-enrichment techniques, called methylation filtration (MF), takes advantage of the difference in methylation between plant genes and repeats [19]. MF exploits the methylation-dependent restriction endonuclease McrBC (modified cytosine restriction) from *E. coli *[20,21]. This enzyme digests DNA in sequences that contain two sites, each one consisting of a purine and a cytosine methylated in carbon 5, separated by 40–3000 bp [22]. Therefore, using an *mcrBC*^+ ^*E. coli *strain as a host to construct a genomic shotgun library, heavily methylated repetitive DNA is efficiently counter-selected, while hypomethylated low copy (*i.e*. genic) sequences are substantially over-represented. MF was first tested in maize, where it yielded a 6-fold enrichment for genes relative to a whole genome shotgun (WGS) library used as a control [19]. Subsequently, MF was applied at large scale in maize [23,24] and in sorghum [25], showing that approximately 95% of the genes in each genome were tagged (A. Chan *et al.*, unpublished) and that most genes and regulatory elements are unmethylated in these two species. These results led to the suggestion that a combination of gene-enrichment and traditional genome sequencing techniques could be combined to efficiently sequence large plant genomes [26]. Further analyses of the large-scale MF data in maize and sorghum also provided insights into the biology of transposable element methylation and activity [23-25]. Pilot MF studies of several monocot, dicot, and non-angiosperm plants (such as pine, fern, and moss) were also conducted [27]. These analyses determined that MF enriches for genes in all plants tested, although to different levels, and that it can be an effective approach to selectively clone and sequence genes from some large plant genomes, where the majority of the DNA is composed of methylated repetitive elements.

In this study we performed a MF analysis of the lycophyte *Selaginella moellendorffii *(family Selaginellaceae), representing a clade not included in previous MF studies. The lycophyte clade diverged from the fern/seed-plant lineage about 400 million years ago [28].

The *S. moellendorffii *sporophyte is diploid and consists of dichotomously branching shoot and root systems. The shoot frequently terminates in arrested buds or bulbils that dehisce and allow clonal propagation. The reproductive structures are the strobili, which form toward the tip of the shoot, each one with either one micro- or megasporangium that produce micro- or megaspores, which in turn germinate and divide mitotically to form either the male or female gametophytes, respectively. The gametophyte produces either motile sperm or egg-forming archegonia. After fertilization of the egg, the new sporophyte remains dependent upon the female gametophyte for a short period of time. *S. moellendorffii *is an excellent model system to study some developmental processes, such as sporogenesis and gametophyte development, which are difficult to study in angiosperms because their spores and gametophytes are dependent upon and surrounded by sporophytic tissues. Seedless plants provide an excellent opportunity to study the epigenetics of these processes, but little is known about DNA methylation and other epigenetic marks in early vascular plants, except for the presence of heterochromatic bands identified by cytological staining [29]. Ferns have been used in attempts to address the methylation of the haploid and diploid generations [30] but their genomes are usually large and only specific sequences were analyzed. The extremely small genome of *S. moellendorffii *(90–130 Mbp; [31]) and its available 8× coverage, high-quality draft genome assembly generated by the Joint Genome Institute of the U.S. Department of Energy (JGI-DOE), will facilitate the study of *S. moellendorffii*'s epigenome and its involvement in the alternation of generations. Due to its small genome size, several transposon families, which are common targets of epigenetic modifications, may be low copy in *S. moellendorffii *and their sequence and epigenetics can be studied without the complications of high copy numbers, allowing the unequivocal identification of individual transposon loci.

Sequences from this study have been deposited in NCBI GenBank under the accession numbers [ET218553–ET221769].

## Results and Discussion

### Sequence data and chloroplast content

**Figure 1:**
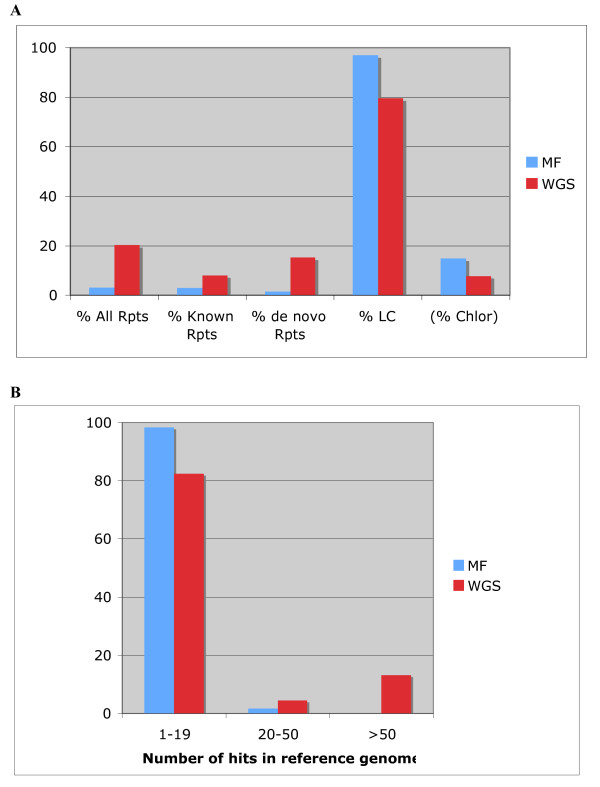
**Proportion of repetitive and low-copy sequences in the MF and WGS libraries**. **A: **Proportion of all low-copy sequences (transcribed, gene-like and anonymous) are shown together (LC). Proportion of all repeats (All Rpts) and their break down into known (Known Rpts) and *de novo *repeats (de novo Rpts) are shown separately. The percentage of chloroplast (Chlor) sequences is calculated relative to the total number of sequences in each library. All the other percentages are calculated relative tot the total of non-chloroplast reads in each library. **B: **Percentages of MF and WGS reads matching the reference genome, classified by the number of hits. Any read showing 20 or more hits in the reference genome is considered a *de novo *identified repeat.

We constructed MF and WGS libraries from *S. moellendorffii *and produced 1,621 and 1,598 high-quality paired sequence reads, respectively, each set representing approximately 1% of the genome. We did not expect a substantial difference in the proportion of gene-like sequences in the MF library relative to the WGS library in the small genome of *S. moellendorffii *because previous studies showed that in the ~400 Mbp genomes of rice and *Ceratodon purpureus *(the smallest genomes in which MF has been tested) the gene enrichment factors (GEF, calculated as the ratio between the MF and WGS proportion of non-repetitive, gene-like sequences) were 1.9 and 2.5, respectively [27]. As the chloroplast genome is typically non-methylated, we prepared total *S. moellendorffii *DNA to construct the MF and WGS libraries in order to retain the chloroplast DNA in both libraries and used it to verify that methylated sequences exist in *S. moellendorffii *and are counter-selected by MF. High-stringency alignments against the *Selaginella uncinata *chloroplast genome [32] identified 14.9% and 7.8% chloroplast DNA sequences in the MF and WGS datasets, respectively (Figure 1A), thus demonstrating that the *S. moellendorffii *genome is methylated and that MF selects for non-methylated sequences as expected.

The chloroplast reads identified in this way were not analyzed further and, therefore, a total of 1,379 MF and 1,471 WGS non-chloroplast reads were used in the following analyses.

Overall, the C+G content is slightly higher in MF than in WGS data (47.9% vs. 46.2, respectively), probably due to the higher C+G content of gene sequences, which are predominant in the MF set (Table 1).

**Table 1:** C+G content in different sequence classes

	%C+G total	%C+G in repeats	% C+G in low-copy DNA	% C+G in genes and EST hits
MF	47.9	50.9	47.8	49.7
WGS	46.2	47.5	45.8	49.6

Approximately 13% of the sequences could not be aligned to the reference genome assembly at the stringency used in this study. This discrepancy may be due to the exclusion of sequence assemblies shorter than 1 kbp from the reference genome sequence.

### Repetitive Sequences

**Figure 2:**
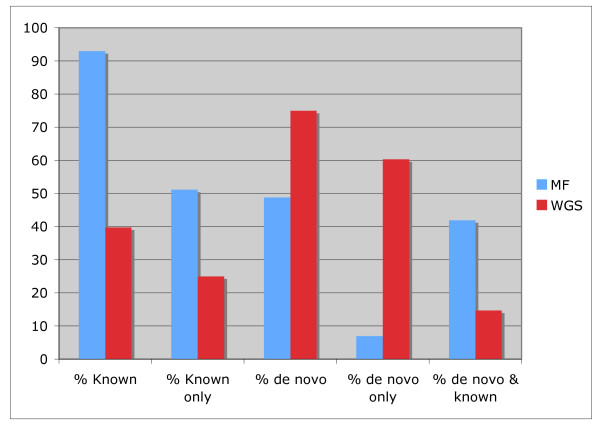
**Proportions of repetitive sequences**. Repeats are classified as matching the repeat databases (Known), identified *de novo*, matching the repeat databases but not identified *de novo *(Known only), identified *de novo *(de novo) but with no match in the repeat databases (de novo only), or that were identified *de novo *and also have a match in the repeat databases (de novo & known). Percentages are calculated relative to the total number of repetitive sequences.

In order to identify repetitive sequences we used nucleotide and amino acid databases of plant repetitive sequences [27]. Consistent with the notion that repetitive elements are methylated in plants, only 2.9% of the MF sequences had a match in either of the repeat databases, while matches in the WGS set reached 8.1% (Figure 1A). As sequences from early vascular plants are underrepresented in available databases, it is possible that many *S. moellendorffii *repetitive elements will not be identified by comparison with previously annotated plant repeats. To better estimate the repeat content of each dataset, we attempted to identify repeats *de novo *by aligning our MF and WGS reads to the draft genome assembly produced by JGI-DOE. Any sequence that had 20 or more high-stringency matches in the reference genome was considered repetitive (Figure 1B and Additional files 1 and 2). A 10-fold reduction in the percentage of these *de novo *repeats was observed in the MF vs. the WGS data set, showing that the highest-copy elements (largest number of hits in the reference genome) are methylated in *S. moellendorffii *(Figure 1A). Taken together, the known and *de novo *repeats account for 3.1% and 20.4% of the MF and WGS reads, respectively (Figure 1A). Among the repetitive MF reads, most were identified as known repeats by database searches, and nearly half were also identified *de novo *(Figure 2), although no MF repeats shows more than 42 copies in the *S. moellendorffii *reference genome sequence, and many are ribosomal RNA sequences (see Additional file 1). Interestingly, all MF sequences matching known transposable elements are low-copy in *S. moellendorffii *(*i.e. *have less than 20 hits in the genome; Additional file 1). In contrast, over 60% of the WGS repeats were identified *de novo*, and do not have a database match, while among those that have similarity to known repeats, nearly 1/3 are high-copy (Figure 2). Furthermore, known WGS repeats show a maximum of 234 hits in the genome, but 1/3 of the WGS repeats have more than 234 copies, the highest having over 500 (see Additional file 2). The prevalence of low-copy repeats detected by MF suggests that low-copy transposons are unmethylated and, therefore, potentially active [6,33,34]. The observed substantial number of WGS unknown high-copy elements highlights the diversity of transposable elements throughout the plant kingdom.

Sequence composition analysis of the repetitive sequences showed that MF repeats are richer in C+G than those in the WGS set, probably due to the abundance of conserved, non-methylated ribosomal RNA sequences among the MF repeats (Table 1).

### Gene sequences, expressed sequences and gene enrichment

**Figure 3:**
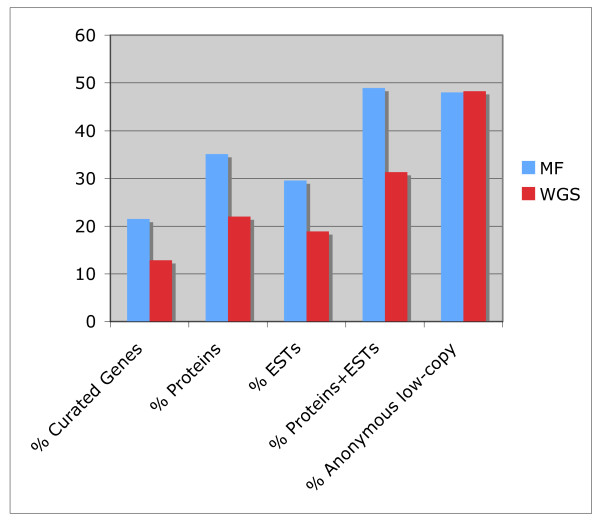
**Gene and transcribed sequence content**. Percentages of matches to the database of curated genes, matches to the NIAA protein database, matches to the *S. moellendorffii *EST assemblies (ESTs), the combination of matches to NIAA protein and EST assemblies databases, and the anonymous low copy-sequences are shown.

Using BLASTX, the MF and WGS non-repetitive sequences were compared to a partially curated, non-identical amino acid sequence database (NIAA) maintained at JCVI, containing most proteins available from GenBank. The percentages of BLASTX matches against this database were 35% and 22% in MF and WGS sequences, respectively (Figure 3), representing a 1.6-fold enrichment in MF relative to WGS sequences, indicating that protein-encoding genes are frequently hypomethylated. Therefore, MF enriches for genes even in the minute genome of *S. moellendorffii*. We also performed high stringency alignments of our sequences to the *S. moellendorffii *assembled ESTs [35], which showed that MF enriches for transcribed sequences to a similar level as it does for protein sequences, suggesting hypomethylation of expressed sequences. Combining the protein and transcribed sequences alignments, 49% of the MF and 31% of the WGS sequences matched either database, while sequences with no database match represented 48% of each dataset (Figure 3).

In order to estimate the level of gene enrichment achieved with MF in *S. moellendorffii *in comparison with previous studies done in other plants [27], all sequences that were not identified as repeats or chloroplast were compared to the same curated database of known gene sequences used in those studies. In this way, 12.8% of the WGS sequences and 21.5% of the MF sequences had a match in the known gene database, resulting in a GEF of 1.7 (Figure 3).

**Figure 4:**
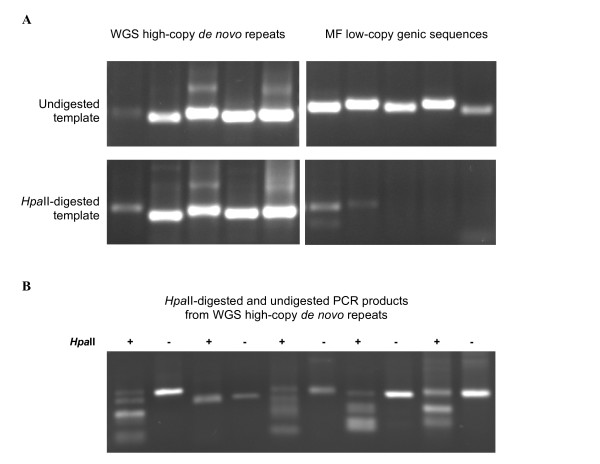
***Hpa*II-digestion and PCR amplificaion of low- and high-copy sequences**. **A: **PCR products ran on agarose gels are shown. Five highly repeated WGS sequences (left panels) and 5 MF sequences (right panels) were amplified from *Hpa*II-digested (bottom panels) or undigested (top panels) *S. moellendorffii *genomic DNA. **B: ***Hpa*II digestion of the 5 PCR products obtained with *Hpa*II-digested genomic DNA from panel A. From left to right, digested and undigested PCR products (in the same order as panel A, bottom left).

We attempted to confirm that the exclusion of high-copy sequences in the MF library was due to DNA methylation using an independent assay. To do this, we digested *Selaginella *genomic DNA with the methylation-sensitive restriction enzyme *Hpa*II, whose restriction target site (CCGG) includes the frequently methylated CpG motif. We then selected 5 of the highest-copy sequences in the WGS library with no match in the databases (*de novo *highest-copy repeats), as well as 5 low-copy MF sequences showing similarity to ESTs and known genes. We designed polymerase chain reaction (PCR) primer pairs so that the expected amplification product would include at least one *Hpa*II site, and carried out PCR reactions with each primer pair using *Hpa*II-digested and undigested DNA as template. The results in Figure 4A show that the amount of amplification product obtained with *Hpa*II-digested template was substantially reduced relative to the undigested control in the 5 low-copy MF sequences, indicating that these sequences are not methylated in the genome, allowing digestion by *Hpa*II and cleavage of the target template sequence. On the other hand, no difference in amplification was observed between the *Hpa*II-digested and undigested high-copy templates. As each of these PCR products correspond to a mixture of sequences from multiple repeated loci, it is possible that some copies of these repeats show variation with respect to the presence of *Hpa*II recognition sites, due to polymorphisms relative to the sequence used for PCR primer design. Thus, the absence of digestion may reflect a lack of *Hpa*II sites or the presence of methylated *Hpa*II sites. To test if *Hpa*II sites were present in the high-copy WGS PCR products amplified from *Hpa*II-digested DNA, we treated these 5 PCR products with *Hpa*II. We observed digestion in all PCR products, indicating that *Hpa*II sites were present and thus, methylated (figure 4B). Nevertheless, we also observed the presence a low proportion of undigested PCR product in addition to the expected digestion products, as well as additional bands, suggesting that multiple polymorphic copies of each repeat were amplified in all cases.

## Conclusion

Our results show that even in the small genome of *S. moellendorffii*, MF sequences display much lower repeat content than WGS sequences, and that each of the identified MF repeats has less than 42 copies in the genome. If the MF repeat sequences are aligned to the reference genome at higher stringency, the number of hits for each repeat decreases, indicating that polymorphisms can be found inside families of repetitive elements (data not shown). Therefore, by sequencing the hypomethylated fraction of the *S. moellendorffii *genome using MF it would be possible to identify which copies of these repetitive elements are methylated. MF of the *S. moellendorffii *genome can be used to obtain information on gene methylation as well, as it has been shown in *Arabidopsis*, where a fraction of the genes do contain cytosine methylation (although at a lower level than repeats and pseudogenes) and this methylation is predominant in particular regions of the genes [14-17]. In consequence, a genome-wide DNA methylation profile can be generated by comprehensive MF sequencing of this genome. Furthermore, combining MF with ultra-high throughput next-generation sequencing techniques will facilitate this kind of analyses using the sequenced genome as a reference. As the variety of *S. moellendorffii *whose genome was sequenced by JGI-DOE has two distinct haplotypes that differ in nucleotide sequence by ~2–5%, (J. Banks, unpublished), it will be possible to determine if there is haplotype-specific DNA methylation using MF sequencing. Genome-wide epigenetic studies of early-diverging land plants will provide the foundation to broaden our understanding of the evolution of epigenetic regulation of developmental processes in plant biology.

## Methods

Total DNA was purified using DNeasy kits (Qiagen, CA) from green tissues of *S. moellendorffii *plants kept in growth chamber. The DNA was mechanically sheared using a Hydroshear device (Genomic Solutions, MI) and fragments ranging from 3 to 4 kb were eluted from an agarose gel after electrophoresis, end-repaired, and ligated into a cloning vector. DNA ligation reactions were transformed into *E. coli *DH5α (*mcrBC*^+^) to consruct the MF library. The WGS library was constructed by introducing the same ligation reaction into *E. coli *GC10 (*mcrBC*^-^). Recombinant clones were sequenced using Big Dye Terminator chemistry and ABI 3730xl sequencers (Applied Biosystems, CA), and vector and low-quality sequences were electronically trimmed.

Chloroplast sequences were identified by BLASTN alignment to the *S. uncinata *chloroplast genome (GenBank accession AB197035) at high stringency (E value smaller than 10^-56^). The chloroplast sequences were excluded from any further sequence analyses. Protein sequence alignments against the NIAA database were done using BLAT. Alignments with at least 70% similarity and 40 amino acids long were recorded as matches.

Alignments to assembled EST sequences were done using BLASTN at high stringency. Matches showing an E value smaller than 10^-56 ^were recorded.

*De novo *repeats were identified by aligning MF and WGS reads to the JGI-DOE *S. moellendorffii *genome assembly using BLASTN and matches covering 50% of the read with 95% identity were recorded.

Alignments to the curated database of known genes were done as previously reported [27], using BLASTX and recording matches with an E value better than 10^-7^.

Known repeats were identified using a nucleotide database and a protein database of known repetitive elements described earlier [27]. These databases do not contain simple sequence repeats. Repetitive element proteins were identified using the protein database of repeats. The same criteria were used to identify known genes, while repetitive nucleotide sequences were identified using BLASTN with an E value smaller than 10^-10^.

DNA digestion with *Hpa*II was preformed following manufacturer recommendations. PCR assays were carried out using 50 ng of *Hpa*II-digested or undigested genomic DNA as template, and denaturing 3 minutes at 94°C followed by 25 amplification cycles using the following program: 30 seconds at 94°C, 30 seconds at 59°C, and 60 seconds at 72°C. Elongation was allowed for 10 minutes at 72°C after amplification. Target and primer sequences are shown in Additional file 3.

## Authors' contributions

APC and HQ performed the sequence analysis; AM–B, DC and SB worked on DNA preparations and library constructions; KO'B performed PCR assays; ML was in charge of sequencing; JAB contributed to the intellectual design of the study; PDR conceived the study, participated in the analysis, and drafted the manuscript. All authors have read and approved the final manuscript.

## Supplementary Material

Additional file 1 **Complete MF analysis results**. An excel file listing BLAST hits of all MF sequences against all databases used, classified as "repeats", "low-copy sequences", and "chloroplast sequences". Click here for file

Additional file 2 **Complete WGS analysis results**. An excel file listing BLAST hits of all WGS sequences against all databases used, classified as "repeats", "low-copy sequences", and "chloroplast sequences". Click here for file

Additional file 3 **PCR primers and target sequences**. A Word document with the selected WGS and MF sequences that were checked by *Hpa*II digestion and subsequent PCR. Primers are shown as underlined sequence and *Hpa*II sites are shown in red. Click here for file
