# Supplementary material for: The highest-copy repeats are methylated in the small genome of the early divergent vascular plant Selaginella moellendorffii
Source: BMC Genomics. 2008 Jun 12;9:282. doi: 10.1186/1471-2164-9-282 (PMC2442089; doi:10.1186/1471-2164-9-282)
Supplement: Additional file 3 — PCR primers and target sequences. A Word document with the selected WGS and MF sequences that were checked by HpaII digestion and subsequent PCR. Primers are shown as underlined sequence and HpaII sites are shown in red. [file 1471-2164-9-282-S3.doc]

>GSLA394TR

TGAGGCAGACGCTGTCTCTACCACAAGTGCCAGTCCATATTCTTGCATGGAACTGCATGG

AGTGTCTGTCACTTGGATGAGGATCCCCATGCATTCTTTTCTCCCACCATCTGTATTGTA

CAGCCTCAGATCCTCGCATTCCATCCCGAAAGTCCAACTGGTGAGCTCCTCCCCCTTCTC

TGCATCCACATTGACAGTGGCTGGAAGAAGAACGCAATTCCTTGCCTGTTCAGAAATGCA

CAACAATTAGAGCTGACAGATGAAAAGCTAGCAGCTGGAAATAACGTGAGGCCACACGCC

ATTCACCACTGCATCAGATGTCTTGAGGCTGCATCCTAAGATGGCGTGAGGCTACACGCC

ATCTGTAACCGAACTGCACTCGAGAGCCCAATCGGTGCATGCCAATGTTGCTACTGAATG

GCATGTGGCCTCATGCCATTTTCTACTTGGCCTAAAAGACTTACCATTTGAAGCCGAATG

GAGGCGTGACAA**CCGG**ACTCGCTGGCGAATGTGCATTGTGTGTGCAGCGGATCGTAGCAG

TAAGGCTACTCTGCCACTTACAGTTGGAGAGTGGTGCTGAAATCTC**CCGG**GACTCCAAGT

GGGCTGCGTGTGGGTGGCGGATTGGGCGGCGGAGTTGGCGGCGAAGTTACAAACTGGGTG

TGTCTTGAAGTGGACGCCGATGCACACGAGCGCGATGCGAGCGCGAGTGTGATGCAAGCG

CGAGTGGACG

>GSLA668TR

GAATGCATGGGGATCCTCATCCAAGTGACAAACACTCCATGCAGTTCCATGCAAGAATAT

GGACTGGCACTTGTGGTAGAGACAGCGTCCGCCTTCAAGATCCACCTTGCACATCCTAGC

GGCTTC**CCGG**AGGGGGGCAGCGGCTATGAGGAATTCCTAGAGGGAGCTAGGGAGAGGTTG

TCCAGGAGGCCCACAATTTCAAAGACGCTG**CCGG**TCTATGAAGATCACATGGAATCCATG

GACCTCACCTACGGCGATATTCAAAAACTATATTCCAACATTGAACCGTTGAGAGTGCCT

CCAGTTCGCGAAGCGAAGGGGAAGGGGGTCGATCTGGAAACTACCCCGAAGCAGAAAAGG

GTGGTCAAGCTCAAGCCGACGTCCGACTCTCCTGCTCCAAAAAAGAAGCTGCTCGGTGAT

GCCATTGCCTCGGGTTCGAACACTACACCCACCAAAAAGAAGAGCCGCAAAGAGCAGAAA

GAGCAAGAAGAGGCAGCAAAAATCGTCGCAGATAATAGTACTTATTACTATTCCAACAAA

GTATATACAATCGACGTAAGCTGTATCGACATCCAAAGCAAATACAACCACAGGCTAGTA

TCTGATGAGTGGGTGGAGAAACTGAA

>GSLA663TR

CAGATGTCTTGAGGCTGCATCCTAAGATGGCGTGAGGCTACACGCCATCTGTAA**CCGG**AC

TGCACTCGAGAGCCCAATCGGTGCATGCCAATGTTGCTACTGAATGGCATGTGGCCTCAT

GCCATTTTCTACTTGGCCTAAAAGACTTACCATTTGAAGCCGAATGGAGGCGTGACAACC

GGACTCGCTGGCGAATGTGCACTGTGTGTGCAGCGGATCGTAGCAGCAAGGCTACTCTGG

CGCTGAAATCTC**CCGG**GACGCCAAGTGGGCTGCGTGTGGGTGGCGGAGTGGGCGGCGGAG

TTGGCGGCGAAGTTACAAACTGGGTGTGTCTTGAAGTGGACGCCGATGCACACGAGCGCG

ATGCGAGCGCGAGTGTGATGCAAGCGCGAGTGGACGCCGATGCACACGAGAAACCTGTAG

AGAAGTGGGAGCGCGAGTGGGAGCGCGATGCGAGCGCGAGTGGGAGCGCGATGCGAGCGC

GAGTGGGAGCGCGATGCGAGCGCGAGTGGGAGCGCGATGCGAGCGCGAGTGTGCGAGCGC

GAGTACGAAGCAAAATGCAAAACCTAACCTCACACGCGCGAAATGTCACGACGGTTGGTA

ATCCCGCCACGTCATCATTTCTCGGACTTGGGCGCCACCATTAATTTCTGGATCGGCGCG

CGCATGCGTGAAATCCAAAAGAAATCTGCAACGTCATCATTCCTCGGACTGCATGCACGC

GAAATCCAAAAGAAATATCATGATTGGGATATCATTTAATACCCTCGCTATATTTATACC

ATGCATGATTGGATATTCCAAAGCATCTTGTATTTCCAAAAATGCCAGATGTTCAGCTAA

ATAAATTGTCAGAGGATTTGATGTGACGTTCAAGCATGTGGGCTCATGCAATTTTGATCC

C

>GSLA578TR

CGGCGGTCGACATCCCTCTTAATGAAATTTGGATACTGATCCAACTTCTCAGCTTTACTT

TTCGTGGCATCCCGCAGCAGATCGATGAACTTGGGGGCCCCCTTCACGTACTCGTCGATG

TCTTCATTTGACAAGTCCAGCTCTTCCATCATCTCTTGCGTGGTGTCGATGCTCACCCTC

TCCACGGCCACTTGCTCGAAGATCCAATCCTTCAATAGCGCACGAGCTCTCAGAGTCGTG

CACAAGTCCTCGTATGTCACAATATGG**CCGG**GAGTCATTTTGGTCTTGTTGAAGACTACC

TCACCACGTAGAAGAGCATTAGCCACTGTCAGAAGGTCGTCGTCGTCTAAAGACCGCGAG

AAACGCAAGGCGTTGAGCTTCAAATCATTGGGCTT**CCGG**CCGTCCTCCACACCCATTGAG

TTTAGCAACTCCCCGTCATGGTACCTCCTCAGCACATCTACCCATTTGTCAAAAACAACT

TTTGTCGCGAATAGAATGTTATGGATGCTGAGTGTGTCATTGACCCCCAGTGT

>GSLA336TF

CCACTGGATCGAGGCCCACAAGCCCGACCACTAAGTCCCACGGTCCTCTGGGACTTCCTC

ACCACACGACTGTGTCCTTGGTCACTACCCCAGAGGACTGCTCCTCTTCCTTTCCACAGT

CTAGGCCTTTGGGACAACATTTCGGAACACCGTGGCCTTACGCGAGCCACCTGGGTACTC

TCCCCGCCTTTCACCTTTAGGTGCTCTAGCCATGGGCTTATAGCGAGGTTTCGTTGAGCC

ACGCCTGGTATCCAAGTAGGTAGTGCCACCCACACTTGGACTGGGAGCATCGGCCAGTCG

ATCTC**CCGG**GGGAACTAGAG**CCGG**CTCTTGAACACTTCACCAAGCTACCCTGCTTGTCGG

TCCGTGTTCACTCTAGTCTTGGACTTCCCAGTGGGCAGCTCATCACCCAACACTGGGTCC

GGCCTTCCTGGGCCAAACTGGCCTTTCCTGGCCAAACTGGCTCCACTGCAGTCCTCCCCC

TACTATACTTTTGGAGCTCACCAAACCCCCCAGGGTCTGGGAGACGAACGCTTTGGGCTT

TCACCCTCTCAGGCT**CCGG**ACTCGGACTCGGACTCGGACTCGGATTCGGACTCGGACTCG

GGCTCGGGCTCGGTCTCGGC

>MFSA305TF

CCTACACT**CCGG**ACGAGCTTCGTGGGAACAGGGTCCGCCGTTCATGAGGAACAGACTTGG

AGGTTGCCTCCCGATGGCATGCGAATCGAACAGCGTTTGCTCTGGGAACGCTCAGTGCAG

CTGTATAGTCCAAGACCAGAGTGGCCATCAGGAGCAAACTTGCCCAAGTCCGAGCAGCAT

ACCTCTCGTTTGCAACCACGAAGTCGAGGCCGAGAACCACCACTTTCTCAACGTGAGTGG

AGCAGGCTACTTCTCCAACAATTACACCAAGGCCGACAGAGTTTCCACTTTGAGCGAGTG

CTGGAGCTTGTGCCTAGCGAATTGCTCCTGCAGCGCACTCTTCTTCAACAAGCGGTCGAG

CACCTGTTTCTTCGTCGA**CCGG**ATGTATGGAGGCCTCACCAGGGATCCCAACTTCGATGG

CTTCCTCAAGCTGCAAAACGCAGAGCTTTTCGTGCGGAAGAAGCCCAAGGATCGCACCGT

TTTGCTTGGAGTCAGCATTGCTGCGAGCGTGGTCCTGCTATCAGCTCTCGGCTTGGTCGT

GCTGATGATCTGGAAGCACAGGCTTGACAAAGTTGAGCGTGCTTTGGCTCTGGCACTGCA

AGGCTCGGCTCAGAAGTATACATACAAGGAGCTGGAGGTGGCGACTGGGAACTTCGCTTC

GAGCCTTGGTAAAGGCGGCTTTGGAACCGTCTATGAAGGGACTCTGGCGGACGGGAGAAA

GGTGGCGGTCAAGCGCCTGGAGAA**CCGG**AACCAGAGTGACAGGGGATTTCTCGCGGAGAT

GGCCTCGCTGGGACGGATAAGCCACCACAACGTTGTCCAGCTCTATGGCTTTTGCGCCGA

GAAGAACCAGCTCATGCTCGTCTACGAGTACGTGGTGAATGGATCCTTGGATAAGTGGCT

GTTCGAAGACCGATGCTTGGGCTGGCAGAGCCGCCGCGATATTGCC

>MFSAA17TF

CCTCGAGTCCGTGTCCTTCATCTTGCGCGCCATCAAGTTGAGGAACTCGGGGAAGTCGAT

GGTGCCGTTGCCGTCCGCGTCCACCTCGTTGATCATGTCCTGGAGCTCCGCCTCGGTCGG

GTTTTGGCCCAGCGATCGCATCACCGTCCCCAGCTCCTTGGTCGTGATGCAGCCTACGAG

AAGAAGAAAAAAATTTGAGTCATGCCCACAAGCTCCAATCGCCCAAATCCCACTCAGGAA

CACACCCATGTCAGCAATGCCATAGATCTGAGCCTCTAACCCTGGGATTCGGGGGCATCA

AACCCTAAATC**CCGG**CTCCAATCCAGATGCCCGCAGC**CCGG**CGCCGCGAATCGATCGATT

CACGAGCTGGACATCACGGAATACCAATGCCAAAGAGAGATTCTCGCACGACAGTGAGGG

ATCAGAGCGCTTACCGTCTCCATCCTTGTCGAAGAGGCTAAACGCCTCCTTGAACTCGGC

GATCTGGTCTTCCGTGAGCTGATCGGCCATCTTCCTCGCGCGCTTCGATGGGCGCCTCCA

CAAAGATCCCTAATCGGTCGCGCCTAGGCTAGATCTCTACTCTCCACGCGTTGATGGCTT

TGACATTAGTTTTACTTTATAGACTATGACATGGCGTCCCAAACTCACGGGAAATATGCC

ACCTCGGACTATACCCTAGTAAAGCCCTAGCAATGGCTGGAGAGGAGGATTTGGCGACGG

CGGAAGCGGAAGGAGACAAGGACGAGCTCGAGGATGAGGAGGAGAAAGAGGTTTGTGAGG

ATCAATTCCAT**CCGG**GCTCGCAAAGCCAGGCTATGGCTCCAGCGATTATCAGCATTCACC

CCAATTCTCGCGCGATTGTGGTCTCAGTGGGAGCTGGATTTCGAGTCTTCGATCTAGAGT

AAGAATTCCGCTCTTGGCGCTTGCGAGGCTGAGTGATTTATTTCCAGGAGCAATGAA

>MFSA483TR

CATAAAAATGAGATTCTGGGACTTGGGCGCTTTGCATTCTTGCTGCCCTGCTCTTGAAAC

ACCTGGAAACGTAAGAAAAACCAGAAATTGGAACTTGGGCGATCGGAATTCTGTGAGAAC

AACACTGATAACTTCGTGTTTTTGCAGTTGGCGATCTCAACCATTTGATCTCGGCGACCA

TGAGCGGCGTGACGTGCTGCCTGCGCTTC**CCGG**GCCAGCTCAACGCCGACCTCCGCAAGC

TGGCCGTCAACCTCATCCCCTTCCCGCGCCTCCACTTCTTCATGATCGGCTTCGCGCCGC

TCACGTCGCGCGGCTCGCAGCAGTACCGCGCGCTCACGGTG**CCGG**AGCTGACGCAGCAGA

TGTGGGACGCCAAGAACATGATGTGCGCCGCGGATCCTCGCCACGGCCGCTACCTCACCG

CCTCGGCGATGTT**CCGG**GG**CCGG**ATGAGCACCAAGGAGGTGGACGAGCAGATGCTCAACG

TCCAGAACAAGAACTCGTCCTACTTCATCGAGTGGATCCCAAACAACGTCAAGTCGAGCG

TGTGCGACATCCCGCCCAAGGGATTGAAGATGTCCGCGACGTTTGTGGGCAACTCCACGT

CCATCCAGGAGATGTTCAAGCGGGTGAGCGAGCAGTTCACGGCCATGTTT

>MFSA384TF

CGGCGTCTCGCGATTTTAAAACTATTC**CCGG**ATCGACGGCTGGGATTGGCCTGCACGGAC

CACAGTGGCCACCGCCCTATTGTTTCAAGCTTTTTGTGTGGGCGACTGCGACAGCGTCGG

GATTATTGGAGGAGGCGATCCATCGATCGATCCATCC**CCGG**GCTCGTCACAGGTCTGATC

GATCGAGCTCTGTGGCTCACATCGCCGCTCTCGCTTAGCGCCCTGTTTCTAGCGAGTTTC

TCCATGGCGGAGGAAGAAATCCAGCCCCTGGTGTGCGACAATGGAT**CCGG**GATGGTCAAG

GTGCGCTCCTGGCGCGGTTTCTTGTGGATTTTGAT**CCGG**GGGGTGTGTTCTTGCAGGCTG

GATTTG**CCGG**AGACGATGCTCCCAGGGCCGTGTTTCCCAGCATTGTGGG**CCGG**CCCAAGC

ACACGGGCGTCATGGTTGGAATGGGGCAGAAGGATGCGTATGTCGGCGACGAGGCGCAAT

CCAAGCGAGGTATCCTGGCATTGAAGTACCCGATCGAGCACGGGATCGTCACAAACTGGG

ACGACATGGAGAAGATCTGGCACCACACTTTCTACAACGAGCTCCGTGTGGCT**CCGG**AAG

AACAC**CCGG**TTCTTCTCACCGAGGCGCCGCTCAACCCAAAGGCGAATCGCGAGAAGATGA

CCCAGATCATGTTCGAGACCTTCAATGTC**CCGG**CCATGTACGTGGCGATCCAAGCAGTCC

TTTCACTCTACGCGAGTGGACGCACTA**CCGG**TATGGAGTTTTTTTATCCCTTGCTTGTTC

TAGACAAGATGGTTGATGCGTGTTTCTTTTCTTCGTTGCTTGCGATAGACATGATGCTTC

AAGTTGGTTCTACTTTTGTTTTTCCCTCTAACACGTTGTATGATCGTGGTTTGTGATCAG

GTATTGTTCTC

>MFSAA74TR

TCTTCTCTACTCCAGCGGTTGGTACTACTGGACGACTTGTCTGTGCTTGGCGATCGCATT

CTTTGCGCTGCTCAATCTCTACTCGAGGAAGAAGGCGCATAGCAATCTTCCAC**CCGG**TCC

CTTTTCCTGGCCTCTTGTTGGATCACTGCCCTCTCTCGGCCGCTCCAGGCACCGCTCTCT

CCATCGTCTCGCCAAGAAGTATGGACCAATCATGTATATGGAGCTGGGAAGCCGTCCTCA

CGTAATCATCAGCAGCCCCGCGATGGCCAGGGAGGTTCTCAAGGATCACGATGTCGAGTT

TGCGAGCAGGCCCCAGTTCCACACCTTCTTCAAGAACGTAAGCCACGACTATAATGATCT

GGTGTTTGCTCCTCACAGCGAGCGCTGGAAGATGTTGCGGCGAGTGAGCGGCACGGAGAT

CTTCGCCG**CCGG**GAGAGTGAACCACTTCTCTGGGATCCGCAAGCGCGAGCTCGCGGCGTT

TGCCTCGATCATCGAGAAGTCGGCGGCTG**CCGG**GGAGGCGTTTGATCTCACCGCGCTGCT

CCACGAGCTCTTCACCAACTTGATGACGTCGGTTCTCTTCGGCCAGAAGTTCTATAACTC

GGACACCCCTGTCACCCGAGAGGCCGAGGCTTATCGCGCTGTGTGGGACACGACCATCGC

GGAGTCGGAGCGGCTGTACATCGGAGACTTCATCCCTGCGCTGCGATGGGTCGACAAGAT

CAAGAGGAGCGAGCACAGGTTGAAGAACCAGATCATA**CCGG**CAGTGGAGAACTTCCTAAA

CGCGATCCTCAAGCAGCACAAGGAGAGCTTCGTCGCGGAGAATCCTCCCAGGGACTTTGT

GGACGTGATGCTCTCACTGGGTGGCGAGAACAAGCTGGACGACATGGCGATCATCGCACT

CCTCCAGGTAAAGGAGAAAAAAATCTCTTCTTGCTCGCTAAGAACTCGTGCTCGATTTCT

TGATAGAATCTTCTCTTAGCTGGGACGAACACTTCCAAAGGCACTGTGGAGTGGGCGATG

TCCGAGATGA
